# Oxidation of Aldehydes Used as Food Additives by Peroxynitrite

**DOI:** 10.3390/antiox12030743

**Published:** 2023-03-17

**Authors:** Clara I. Alcolado, Luis Garcia-Rio, Juan C. Mejuto, Inmaculada Moreno, Francisco J. Poblete, Juan Tejeda

**Affiliations:** 1 Department of Physical Chemistry, Faculty of Chemistry, University of Castilla—La Mancha, Avda. Camilo José Cela s/n, 13071 Ciudad Real, Spain; 2 Department of Physical Chemistry, Faculty of Chemistry, University of Santiago, Avda. Das Ciencias s/n, 15701 Santiago de Compostela, Spain; 3 Department of Physical Chemistry, Faculty of Science, University of Vigo, Campus de As Lagoas, 32004 Ourense, Spain

**Keywords:** oxidation, aldehydes, peroxynitrite, food additives, organoleptic properties, antioxidant properties

## Abstract

Benzaldehyde and its derivatives are used as food supplements. These substances can be used mainly as flavorings or as antioxidants. Besides, peroxynitrite, an oxidizing agent, could be formed in canned food. Both species could react between them. The present article has focused on the kinetic study of the oxidation of aldehydes by peroxynitrite. A reaction mechanism that justifies all the experimental results is proposed. This mechanism, in acidic media, passes through three competitive pathways: (a) a radical attack that produces benzoic acid. (b) peracid oxidation, and (c) a nucleophilic attack of peroxynitrous acid over aldehyde to form an intermediate, X, that produces benzoic acid, or, through a Cannizzaro-type reaction, benzoic acid and benzyl alcohol. All rate constants involved in the third pathway (c) have been calculated. These results have never been described in the literature in acid media. A pH effect was analyzed.

## 1. Introduction

Benzaldehyde (BZH) is a substance mainly used as a food supplement because of its organoleptic activity, such as cinnamon flavoring. Moreover, this product presents a high level of antioxidant activity [1,2]. Peroxynitrite (HOONO/ONOO^−^) is present in cells and animal tissues. Its formation is mainly observed in canned meats, fruits, and vegetables due to anaerobic conditions. Thus, peroxynitrite can not only act as an antioxidant but also as a pro-oxidant [3,4]. Both compounds can react with each other when they meet. Then, peroxynitrite would oxidize the aldehyde present in food or food additives, thus altering their physicochemical properties. The control of these degradation/oxidation processes is therefore of great interest to the food industry. The obtained reaction products show, depending on the concentration, high levels of toxicity.

More than 300 aldehydes have been identified in more than 300 different foods or food components. More specifically, benzaldehyde is found in 150 of those 300 different foods or food components, such as alcoholic beverages, dairy products, meat, fruits, vegetables, coffee, tea, cocoa, and cinnamon [5]. According to Krings et al. [6] benzaldehyde is considered the second most important food flavoring agent. It is a key ingredient in natural fruit flavorings. Benzaldehydes appear on the list of Generally Regarded as Safe (GRAS) flavoring substances in Europe [7].

On the other hand, the importance of peroxynitrite chemistry is focused on biological systems, specifically in aerobic organisms’ cells, that continuously produce pro-oxidant reactive species formed from different metabolic processes as free radicals. The most important detrimental effect that free radicals can cause in the cells is the generation of so-called nitrosative/oxidative stress [8]. The production of peroxynitrite occurs when there is an overproduction of radicals derived from oxygen/nitrogen or when there is a deficiency in antioxidant mechanisms. As a result, the excess of radicals can permanently damage the different macromolecules present in the cell, such as proteins, lipids, carbohydrates, and nucleic acids [9]. Peroxynitrite can produce disruption of cellular structures, inactivation of enzymes and ion channels by oxidation and nitration of proteins, and DNA damage [10].

The chemistry of peroxynitrite under physiological conditions has been extensively studied in the biomedical field due to its formation/reaction in oxidative/nitrosative stress [11,12]. In contrast, bromatology has not been so well developed. This research in this field is therefore of great interest in understanding the action mechanism of peroxynitrite with different foods or their additives, in which it can induce oxidative reactions. These reactions can modify the physicochemical properties of canned food. There are two ways to form a peroxynitrite precursor species (NO) in biological tissues [13]:

1. The main pathway to produce nitric oxide is through nitric oxide synthase in uncured meats. This enzyme reduces its activity due to low pH, low-temperature storage, salting, and/or thermal processing operations typical for muscle foods.
2. Postmortem calcium loss can stimulate nitric oxide synthase activity, increasing nitric oxide formation and creating favorable conditions for peroxynitrite production.

In plant tissues, peroxynitrite will be produced when lipid oxidation occurs, mainly caused by injuries due to chilling, wounds, SO_2_ fumigation, ozone exposure, high salt concentrations, or dehydration [13].

Once formed, peroxynitrite can affect the oxidative stability of foods, leading to the appearance of rancidity, loss of color, and alterations in the functional properties of proteins, decreasing the quality of the food [14]. This oxidation process can be modified by changes in pH, salt, carbon dioxide concentration, and different processing and storage temperatures [13].

Thus, peroxynitrite can act as a radical former [15] or as a hydroxylating, nitrosating agent, and oxidizing agent due to its redox potential, 1.4 V at pH = 7, one of the strongest that has been studied [16]. As a novelty, in this study, peroxynitrite presents reactivity as a nucleophilic organic species. To analyze which are the reactive chemical species and their role in the reaction mechanism, the decomposition of peroxynitrite must be considered. In this way, Koppenol et al. [17] have proposed a decomposition mechanism that implies two ways: the formation of radicals and the production of one activated intermediate. The degree of radical formation is not fixed for all reactions involving peroxynitrite, but rather depends on the reaction conditions, the characteristics, and the concentration of the oxidizable substrates [18,19,20].

The purpose of this study is to know in detail the different benzaldehydes’ decomposition processes in the presence of peroxynitrite in order to analyze both at different concentrations, which are similar to those found in food, with concentrations ranging from 0.5 mM to 0.04 mM present in both cells and food [21,22,23]. In addition, a quantitative structure-activity relationship (QSAR) was analyzed for different substituted benzaldehydes to confirm the proposed mechanism. The chosen aldehydes are present as flavorings in canned meat, fruit, vegetables, alcoholic and non-alcoholic beverages, candies, ice creams, jellies, cheeses, pastries, etc. [24]. Thus, the selected ones were 4-hydroxybenzaldehyde (pHBZH), 4-methylbenzaldehyde (pMeBZH), 4-methoxybenzaldehyde (pMoxBZH), 4-nitrobenzaldehyde (pNBZH) and 4-trifluoromethylbenzaldehyde (pTFBZH).

In advance, benzaldehydes are oxidized by peroxynitrite through a mechanism formed by three competitive pathways: a radical attack, peracid oxidation, and a nucleophilic attack in acidic media that ends in a Cannizzaro reaction type, described in acidic media for the first time in this work.

A bibliographic review [25,26,27,28] shows that both the radical and the oxidative mechanisms have been studied for aldehydes and other organic compounds, but the nucleophilic attack in an acid medium has not been studied. For them, this study is centered on this nucleophilic attack.

The goal of this work is to analyze for the first time in an acid medium a Cannizzaro-type mechanism present in the reaction as well as the study of QSAR. In order to provide sufficient chemical knowledge through the reaction mechanism to support the storage industrial process of different foods in which the molecules in this study are present.

## 2. Materials and Methods

### 2.1. Reagents

#### 2.1.1. Peroxynitrite Synthesis

The peroxynitrite synthesis used in this work was described [29]. This method was selected because it improves the yield (almost 100%) and does not generate species that modify the initial reaction rate.

The reaction must be carried out in a basic medium with equimolecular amounts of H_2_O_2_ since nitrites (NO_2_^−^) are highly unstable in both neutral and acidic media. This way, the final hydrogen peroxide concentration would be negligible. The obtained products are peroxynitrite and 2-ethoxyethanol. Although the last one can react with ONOO^−^, this reaction is very slow.

The experimental procedure is as follows:

- Synthesis of 2-ethoxyethylnitrite: 2.78 mL of sulfuric acid 2M are added dropwise to a 12 mL of ethoxyethanol 2.5 M solution with 40 gr sodium nitrite at 0 °C. The reaction finishes when no more nitrogen oxides are released. After an hour, the alkyl nitrite phase is refrigerated in an opaque container with 2 mm molecular sieves.
- Synthesis of peroxynitrite anion: Once peroxynitrite is prepared by mixing 0.2 mL of 2-ethoxyethylnitrite (precursor) with 15 mL of hydrogen peroxide 0.109 M and 15 mL of sodium hydroxide 2 M in 70 mL of H_2_O. The resulting anion must be stored at −18 °C. The peroxynitrite concentration is determined daily by spectrophotometry at 302 nm.

#### 2.1.2. Other Reagents

The rest of the reagents used during this work have “for analysis” purity, and all the solutions are prepared with double-distilled water. All reagents were purchased from Sigma Aldrich (Madrid, Spain).

### 2.2. Procedure and Measurements

The experimental results were obtained when two solutions (A and B) were introduced into a Stopped Flow SX-20MV spectrometer from Applied Photophysics (Leatherhead, UK) equipped with an anaerobic kit. This kit avoids the presence of gases such as O_2_ (a radical inhibitor) and CO_2_ (which forms reactive complexes) within the reaction. The solutions were mixed by 50%.

The first solution A contains the required concentrations of benzaldehydes, ionic strength Na_2_SO_4_, and H_2_SO_4_. The second one, B, contains the necessary concentration of peroxynitrite in diluted sodium hydroxide and Na_2_SO_4_. Both solutions were previously bubbled with Argon for 20 min in flasks or Schlenk tubes for the complete elimination of dissolved CO_2_ and O_2_. Table 1 shows the concentration range used in the experimental procedure.

The pH values were measured at the beginning and end of all kinetic experiments by using a Crison micropH 2000 pH meter (Barcelona, Spain). The observed pH values are kept constant. Organic buffers have not been used because peroxynitrite is capable of reacting with most organic buffers.

All the experiments were performed in quintuplicate to reduce errors.

The control chemical species were benzaldehydes, and their concentrations were measured at the different maximum absorption wavelengths. For this analysis, UV-visible spectra were recorded with a Varian Cary 50 UV-visible spectrophotometer (Santa Clara, CA, USA). Table 2 shows the wavelength of absorption maximum together with its molar absorptivity coefficients for each one of the reagents.

The molar absorptivity coefficients of all the aldehydes under study are considerably higher than the molar absorptivity coefficient of peroxynitrite; therefore, despite the proximity of their absorption maxima, any overlap of the bands is negligible compared to the value of this parameter. This fact confirms its choice as a control chemical species.

The pH of the study was checked at the beginning and end of all kinetic experiments by using a Crison micropH 2000 pH meter (Barcelona, Spain).

### 2.3. Kinetic Analysis

The experimental data were analyzed using the initial rate method. This method avoids the following problems:

- Product interference;
- Self-decomposition of reactants;
- Inhibition or autocatalysis effects;
- Presence of competitive reactions.

**Figure 1:**
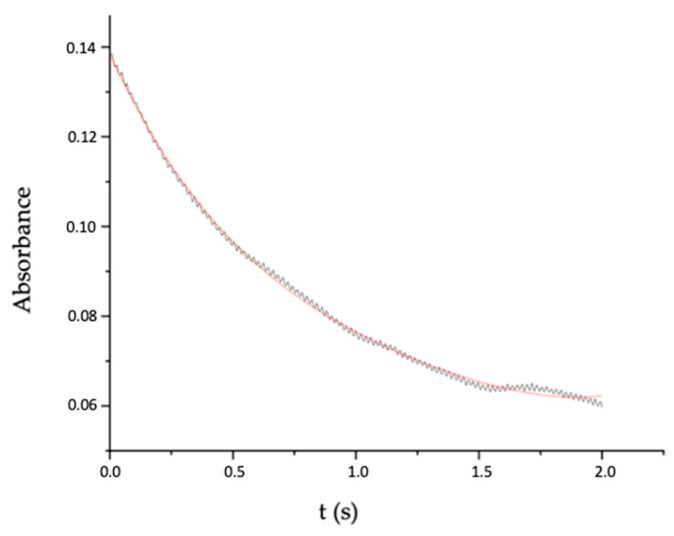
Absorbance vs. time plot, [p-HBZH] = 8.00 × 10^−5^ M, [HOONO] = 2.00 × 10^−3^ M, T = 25 °C, pH = 2, I = 0.1 M.Black line: experimental data. Red line: curve fit to the Equation (1).

The collected absorbance-time data are fitted to a four-degree polynomial [30] and analyzed statistically in the Origin2019b program (OriginLab Corp., Northampton, MA, USA). The fittings were made for at least 2 s of the absorbance data against the reaction time of the kinetic experiment, obtaining about 40–50% of the reaction. For example, Figure 1 shows absorbance/time data for the p-hydroxybenzaldehyde reaction, these data were fitted to Equation (1) (red line) (r = 0.9989)

(1) $$A=a+\mathrm{bt}+{\mathrm{ct}}^{2}+{\mathrm{dt}}^{3}+{\mathrm{et}}^{4}$$

In order to determine the initial reaction rate, the Lambert-Beer law should be considered. Deriving Equation (1) with respect to time and in account that t = 0 can be written as:

(2) $$v_{o}=-{\left(\frac{d\left[\mathrm{RBZH}\right]}{\mathrm{dt}}\right)}_{0}=-\frac{1}{\varepsilon \cdot l}\cdot b$$

The experimental values and errors obtained in this kinetic study represent an arithmetic mean of five reactions carried out under the same experimental conditions to ensure the reproducibility of the study.

## 3. Results

In the next paragraphs, benzaldehyde will be used as an example to explain the experimental results; the rest of the data for the other aldehydes studied are included for consultation in the Supplementary Materials.

### 3.1. Influence of Substrate and Oxidant Concentrations on the Reaction Rate

A series of experiments were programmed to analyze the influence of substrate concentration on the reaction rate, in which only benzaldehyde concentration was varied, keeping pH, ionic strength, and temperature constant.

**Figure 2:**
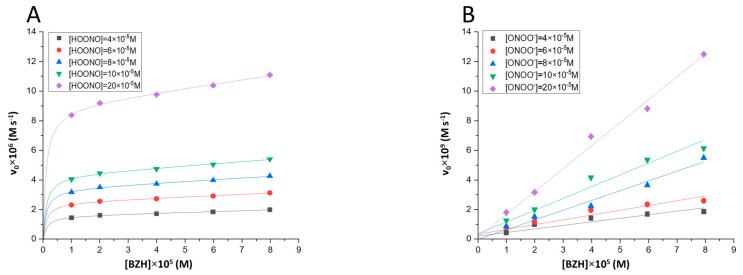
Influence of substrate concentrations on initial reaction rate. Experimental conditions: I = 0.1 M, T = 25 °C, (**A**) pH = 2, (**B**) pH = 11.2.

The results are shown in Figure 2. This figure shows on the left the graph corresponding to the experimental data in an acid medium and on the right those corresponding to a basic medium. 

As can be seen in the curves of Figure 2 at a pH of 2, it is observed that the initial speed increases a lot initially and then grows to a lesser extent without stabilizing. The mathematical equations that agree with this variation are rational polynomial equations of at least degree two in the numerator and denominator. All the possibilities were checked using the variance analysis (ANOVA) method. To analyze in depth these equations a test p-F was employed among other statistical parameters. The closer to zero the value of the parameter p-F is, the better the mathematical fit will be. The lower the p-F, the better the fit, which is shown in Equation (3).

(3) $$v_{0}=\frac{A{\left[\mathrm{RBZH}\right]}_{0}+B{\left[\mathrm{RBZH}\right]}_{0}^{2}}{1+C{\left[\mathrm{RBZH}\right]}_{0}}$$

This equation would imply the formation of an intermediate complex (X), which would be attacked by another substrate molecule.

For pH = 11.2, the same analysis (ANOVA) was performed, and the p-F was used as a differentiating statistical parameter, thus giving the best fit to a linear fit, Equation (4).

(4) $$v_{0\;}=E\;\left[\mathrm{RBZH}\right]$$

### 3.2. Influence of Peroxynitrite Concentration on the Initial Reaction Rate

**Figure 3:**
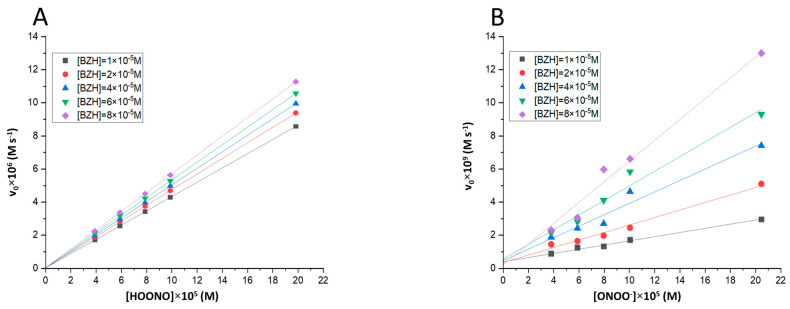
Influence of peroxynitrite concentration on the initial reaction rate. Experimental conditions: I = 0.1 M, T = 25 °C, (**A**) pH = 2, (**B**) pH = 11.2.

To perform this analysis, a series of experiments were carried out where only the peroxynitrite concentration was varied. The results are shown in Figure 3. This figure shows that, for acidic and basic media, the variation of the initial rate with respect to peroxynitrite concentration is a straight line through the origin. The best mathematical fit obtained were:

At pH= 2

(5) $$v_{0}=D{\left[\mathrm{HOONO}\right]}_{0}$$

At pH= 11.2

(6) $$v_{0\;}=G{\left[{\mathrm{ONOO}}^{-}\right]}_{0}$$

### 3.3. Influence of pH on the Initial Reaction Rate

In order to study the effect of pH on the initial reaction rate, a series of experiments were carried out in which benzaldehyde (4.00 × 10^−5^ M) and peroxynitrite (8.00 × 10^−5^ M) concentrations, ionic strength (0.1 M) and temperature (25 °C) were kept constant, varying the pH value from 1 to 12, where the best mathematical fit was:

(7) $$v_{0}=\frac{J\left[H^{+}\right]+K{\left[H^{+}\right]}^{2}}{1+L\left[H^{+}\right]}$$

**Figure 4:**
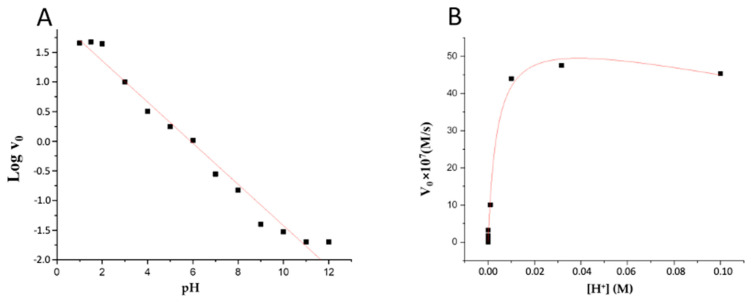
Influence of pH on the initial reaction rate. [HOONO]= 8.00 × 10^−5^ M, [BZH]= 4.00 × 10^−5^ M, I = 0.1M, T = 25 °C. (**A**) Specific acid catalysis. (**B**) initial rate versus [H^+^].

These results can be observed in Figure 4. In this figure, the initial reaction rate increases with increasing proton concentration, abruptly reaching a maximum, from which the reaction rate decreases slightly with increasing proton concentration.

Although a straight line is observed when plotting log v_0_/pH in acidic media, the slope presents a value of −0.347 ± 0.001 (r = 0.9771), and the specific catalysis is not considered because the slope differs greatly from −1.

### 3.4. Influence of the Ionic Strength on the Initial Reaction Rate

No effect of ionic strength was observed, so at least one of the species involved in the velocity determining step is neutral. The effect of ionic strength on the kinetic coefficients depends on the magnitude and sign of the charges of the reactants [31]. By applying the extended Debye-Hückel law, it can be obtained: [32], (Equation (8)).

(8) $$\log\;\left(\frac{v}{v_{0}}\right)=2{\;z}_{A}z_{B}\left(\frac{A\sqrt{I}}{1+\sqrt{I}}\right)$$

### 3.5. Influence of Temperature on the Initial Reaction Rate

Moreover, the influence of temperature on the initial reaction rate was analyzed in the range between 10 and 30 °C. It is observed in Table 3 that the initial reaction rate increases with temperature.

### 3.6. Product Determination

The technique selected for the determination of products was proton nuclear magnetic resonance spectroscopy, ^1^H-NMR.

For product determination, 25 experiments were performed under kinetic conditions with the following characteristics: [RBZH] = 4.00 × 10^−5^ M, [HOONO] = 8.00 × 10^−5^ M, I = 0.1 M, pH = 2 and 11.2, 25 °C. The reaction was stopped after 15 min by adding ethyl ether to extract the organic products. The organic phase is dried with magnesium sulphate, filtered, and then the solvent is removed.

The products were determined by ^1^H-NMR. In addition to benzaldehyde, the presence of benzoic acid and benzyl alcohol were detected in the acidic medium and only benzoic acid in the basic medium.

## 4. Discussion

The proposed mechanism that could justify all the experimental results must consider the peroxynitrite decomposition [17] as a source of reactive species: peroxynitrous acid, peroxynitrite anion, and radical species such as ·NO_2_ and ·OH. Thus, these species must be considered to react with benzaldehyde. 

Moreover, the presence of benzyl alcohol justifies the proposal of a Cannizzaro-type reaction in acidic media.

**Scheme 1:**
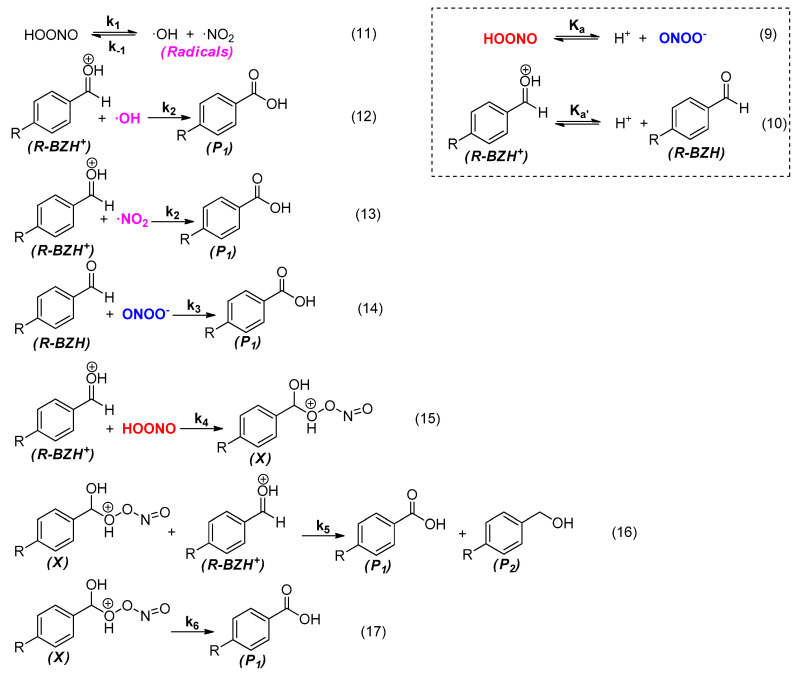
Reaction mechanism.

Considering these species as well as the mathematical adjustments of the experimental results, it is proposed the reaction scheme; see Scheme 1 (the detailed mechanism can be found in the supporting information).

The reaction mechanism begins with the formulation of acid-base equilibria for peroxynitrite (9) and benzaldehyde (10).

Subsequently, three competitive stages are described:

- Firstly, the oxidation of the substrate by the radicals formed from peroxynitrite decomposition (11), (12), and (13).
- The second is the direct oxidation of the aldehyde by the peroxynitrite anion (14).
- The third is the nucleophilic attack of peroxynitrous acid on benzaldehyde to form an X species that can form benzoic acid and benzyl alcohol (a Cannizaro-type reaction).

The first two stages have been studied for other aldehydes in the literature [26,33], while the third one has never been described.

Due to its novelty, this work is focused on the Cannizaro-type reaction, which involves a nucleophilic attack of peroxynitrous acid over the protonated aldehyde to form the X adduct (15). This species can either decompose to yield benzoic acid (17) or react with another benzaldehyde molecule to produce benzoic acid and benzyl alcohol (16).

In this way, the main product observed experimentally was benzoic acid, which generates the rancidity of the added additive, altering its organoleptic properties, and, in high concentrations, can be harmful to health.

The theoretical rate equation obtained from the proposed mechanism is as follows:

(18) $$\begin{array}{l} v_{0}=-\frac{d\left[\mathrm{RBZH}\right]}{\mathrm{dt}}=\{k_{2}\left[{\mathrm{RBZH}}^{+}\right]\left[\cdot \mathrm{OH}\right]+k_{2}\left[{\mathrm{RBZH}}^{+}\right]\left[\cdot {\mathrm{NO}}_{2}\right]\}+k_{3}\left[\mathrm{RBZH}\right]\left[{\mathrm{ONOO}}^{-}\right]+\{k_{4}\left[{\mathrm{RBZH}}^{+}\right]\left[\mathrm{HOONO}\right]+\\ +k_{5}\left[{\mathrm{RBZH}}^{+}\right]\left[X\right]\} \end{array}$$

In this equation, three terms that correspond with the three competitive pathways are observed.

– The first one is the oxidation of the substrate by the radicals formed by peroxynitrite decomposition (11), (12), and (13) from the mechanism. As [·OH] = [·NO_2_] and presents a similar reactivity, the equation $\left\{k_{2}\left[{\mathrm{RBZH}}^{+}\right]\left[\cdot \mathrm{OH}\right]+k_{2}\left[{\mathrm{RBZH}}^{+}\right]\left[\cdot {\mathrm{NO}}_{2}\right]\right\}$ can be written as follows:
  (19) $$v_{0\;\mathrm{rad}}=2k_{2}\left[{\mathrm{RBZH}}^{+}\right]\left[\mathrm{Rad}\right]$$
– The second one is the direct oxidation of the aldehyde by the peroxynitrite anion (14) from the mechanism, which is described in the theoretical rate equation as follows:
  (20) $$v_{0\;\mathrm{ox}}=k_{3}\left[\mathrm{RBZH}\right]\left[{\mathrm{ONOO}}^{-}\right]$$
– The third one is the nucleophilic attack of peroxynitrous acid over de-protonated aldehyde that increases carbonyl carbon’s electrophilicity to form X. X can decompose or can react through a Cannizzaro-type. Thus, it can be written as:
  (21) $$v_{0\;\mathrm{Nu}}=\left\{k_{4}\left[{\mathrm{RBZH}}^{+}\right]\left[\mathrm{HOONO}\right]+k_{5}\left[{\mathrm{RBZH}}^{+}\right]\left[X\right]\right\}$$

Applying the steady state theory to the [X] adduct and considering the mass balance equation with respect to peroxynitrite, it can be written:

(22) $$v_{0}=\frac{2k_{4}k_{5}A{\left[\mathrm{RBZH}\right]}^{2}{\left[H^{+}\right]}^{2}+k_{4}k_{6}k_{a}^{\prime }A\;\left[\mathrm{RBZH}\right]\left[H^{+}\right]}{k_{a}^{\prime }k_{5}\left[\mathrm{RBZH}\right]\left[H^{+}\right]+k_{6}k_{a}^{\prime }{}^{2}}\;{\left[\mathrm{HOONO}\right]}_{0}$$

where A:

(23) $$A=\left(\frac{\left[H^{+}\right]}{\left[H^{+}\right]+K_{a}}\right)$$

To evaluate the reaction percentage that follows each one of the three competitive stages, a free radical scavenger was used. Hydroquinone, known for its efficiency as a radical inhibitor [34], was added until no variation of the initial rate was observed, resulting in approximately a 3:1 ratio of [hydroquinone]/[peroxynitrite]. When this fact occurs, the radical pathway is completely inhibited, so that the reaction percentages for each competitive stage can be calculated as shown in Table 4.

As can be seen in Table 4, in the acidic medium, mainly two of the three mechanism stages appear: radical and nucleophilic attacks, which are in accordance with the concentration of peroxynitrite anion present [OONO^−^] = 2.5 × 10^−5^ [HOONO]. This last stage does not have a specific acid catalysis since it depends on the concentration of protons squared (Equation (22)). On the contrary, in basic media, radical attack and direct oxidation are more important, which do not present acid catalysis.

At pH = 2, the percentage of the radical pathway is similar to that mentioned in the bibliographical data [15]. Taking these results into account, certain rate constants can be evaluated as k_3_ = 0.717 ± 0.005 M^−1^ s^−1^ (oxidation process) and the constants involved in the nucleophilic attack. Table 5 shows these k_4_ calculated values, related to nucleophilic attack to form X, and k_5_, the reaction of X with another aldehyde molecule, and k_6_, which corresponds to X decomposition.

In Table 5, it can be observed that k_5_ and k_6_ increase as σ decrease, that is when activating groups appear. If k_5_ and k_6_ are compared for each of the substituted benzaldehydes, it can be observed lower σ, reaction governed by k_6_ becomes more relevant.

To know the aromatic substituents, and their influence upon nucleophilic attack, the reactivity-structure correlation was analyzed using Hammett Equation (24) [36]. For this purpose, aldehyde nucleophile addition constants in acid medium, k_4_ values versus σ were plotted.

(24) $$\log\;\left(\frac{k}{k_{0}}\right)=\sigma \rho$$

**Figure 5:**
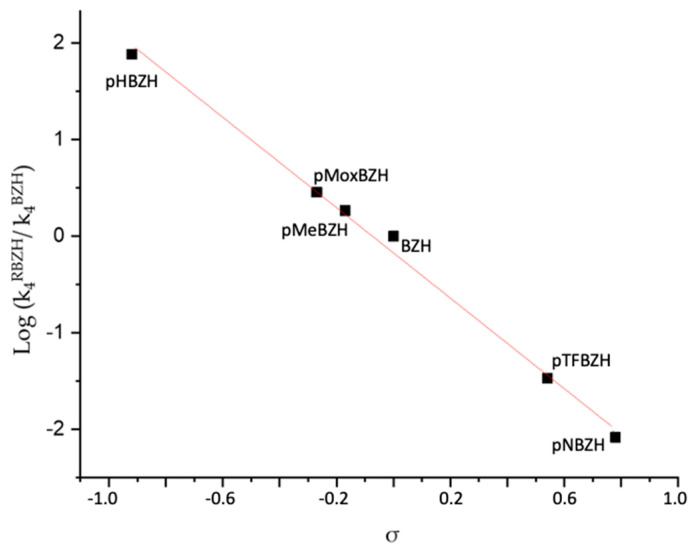
Graphical representation of the Hammett equation for k_4_, nucleophilic attack.

These results are shown in Figure 5. As σ increases, peroxynitrous acid nucleophilic addition to the benzaldehydes aldehydic group rate constant, k_4_, decreases.

The obtained Hammett ρ parameter value was −2.34 ± 0.01 (r = 0.9951). Negative values imply that a positive charge is being generated (or a negative charge is lost) since for benzaldehydes with donor substituents such as –OH, –OCH_3_, or –CH_3_, higher values of k_4_ have been obtained, while for the attractor substituents (–NO_2_ and –CF_3_) the opposite occurs. This justifies the stabilization of a positive charge in the transition bay donor substituents.

In short, depending on the used benzaldehydes’ structure, greater or lesser benzoic acid derivatives amount will be produced (a substance that should be a minority product in foodstuffs). Donor substituents increase the production of benzyl alcohol and decrease the production of benzoic acid.

## 5. Conclusions

Aldehyde additive degradation transforms its organoleptic properties and produces toxic benzoic acid derivatives. This is the main reason why this work has been focused on the study of this oxidation/degradation process.

Considering the obtained results, three simultaneous reaction pathways have been proposed to justify the experimental data: a radical attack, a direct oxidation by the peroxynitrite anion that produces benzoic acid, and finally a nucleophilic attack through a Cannizzaro-type reaction that yields benzoic acid and benzyl alcohol. This mechanism has never been described in literature for acid media. This nucleophilic attack of peroxynitrous acid on the carbonylic carbon is enhanced since the aldehyde group is protonated. The amount of toxic acid could be reduced by increasing the k_6_ constant of the third pathway, as can be observed in Table 5.

On the other hand, the Hammett correlation proves that the presence of activating groups can favor the formation of the adduct X and reduce acid production.

Consequently, these factors must be considered for better organoleptic property conservation and, therefore, being able to avoid food rancidity and the appearance of undesirable toxic products. It is not easy to implement this kinetics data at an industrial level because, in view of this study, aldehydes have two functions, one organoleptic or sensory, and the other is their activation as antioxidants. This dual function presents problems when substances harmful to health are produced, such as benzoic acid and benzyl alcohol. By altering the experimental conditions or substituents of benzaldehyde, the appearance of these products can be modified.

Therefore, what must be avoided is the appearance of peroxynitrite in any preservative type since this molecule is a very strong oxidant and, as can be seen in this study, at a basic pH the chemical attacks of this molecule are reduced under anaerobic conditions ($v_{0\;\mathrm{pH}=2}=2.48\times 10^{-6}\;M/s\;,{\;v}_{0\;\mathrm{pH}=11.2}=2.46\times 10^{-9}\;M/s$). It should be considered that the use of a greater number of chemical controls, such as the changing of the products used in the conservation industry, can increase costs. Therefore, an exhaustive control of the pH in each preservative produced by the industry would be very useful.

## Figures and Tables

**Table 1:** Concentration range used in the experimental procedure.

Conditions	Benzaldehydes	Peroxynitrite	pH	Na_2_SO_4_	T
**Range**	(1.00–8.00) × 10^−5^ M	(4.00–20.00) × 10^−5^ M	2–12	0.1 M	25 °C

**Table 2:** Absorption maxima and molar absorptivity coefficients of the studied species. T = 25 °C.

Reagent	λ (nm)	ξ (M^−1^ cm^−1^)
Peroxynitrite (HOONO/ONOO^−^)	302	1682 ± 11
Benzaldehyde (BZH)	250	12,526 ± 128
4-Hidroxybenzaldehyde (pHBZH)	285	15,762 ± 67
4-Methylbenzaldehyde (pMeBZH)	280	12,648 ± 118
4-Metoxybenzaldehyde (pMoxBZH)	282	20,490 ± 187
4-Nitrobenzaldehyde (pNBZH)	270	12,771 ± 55
4-TrifluoroBenzaldehyde (pTFBZH)	240	11,482 ± 119

**Table 3:** Influence of temperature over the initial rate. [HOONO] = 8.00 × 10^−5^ M, [BZH] = 4.00 × 10^−5^ M, I = 0.1 M, pH = 2.

**Temperature (K)**	283	288	293	298	303
**v_0_** × **10^6^ (M·s^−1^)**	0.82 ± 0.02	1.52 ± 0.02	2.41 ± 0.02	3.82 ± 0.02	5.27 ± 0.02

**Table 4:** Percentage of mechanism routes.

	pH = 2	pH = 11.2
**Radicals**	35%	3%
**Oxidation**	-	97%
**Nucleophilic attack (Cannizzaro)**	65%	-

**Table 5:** Reaction constants for the aldehydes studied, together with the bibliographic values obtained for the parameter σ [35].

Aldehyde	k_4_ × 10^−3^ (M^−1^s^−1^)	k_5_ (M^−1^s^−1^)	k_6_ × 10^+14^ (s^−1^)	σ
**BZH**	24.37 ± 1.21	14.29 ± 0.71	1.06 ± 0.05	0.00
**pHBZH**	1861.42 ± 93.07	6298.23 ± 314.91	2,300,000 ± 115,000	−0.92
**pMeBZH**	44.86 ± 2.24	75.14 ± 3.75	172 ± 8.6	−0.17
**pMoxBZH**	69.53 ± 3.47	340.31 ± 17.01	5450 ± 272.5	−0.27
**pNBZH**	0.20 ± 0.01	0.56 ± 0.02	0.01 ± 0.0005	0.78
**pTFBZH**	0.82 ± 0.04	1.69 ± 0.08	0.05 ± 0.002	0.54

## Data Availability

The data are contained within the article and Supplementary Materials.

## Supplementary Materials

The following supporting information can be downloaded at: https://www.mdpi.com/article/10.3390/antiox12030743/s1, Table S1: Initial rates of all aldehydes in both media; pH = 2; I = 0.1 M; T = 25 °C. Table S2: Influence of pH on the initial reaction rate. [RBZH] = 4.00 × 10^−5^ M; [ONOO^−^] = 8.00 × 10^−5^ M; I = 0.1M; Tª = 25 °C. Table S3: Initial velocity values obtained at different ionic strength values at pH = 11.20 and pH = 2. [BZH] = 4.00 × 10^−5^ M; [ONOO^−^] = 8.00 × 10^−5^ M. Table S4: Influence of temperature on the initial rate [HOONO] = 8.00 × 10^−5^ M. [BZH] = 4.00 × 10^−5^ M. I = 0.1 M. pH = 2. Table S5: pK’a values for each of the studied benzaldehydes. Table S6: Parameters of the rational fit and reaction constants obtained for each aldehyde. Table S7: Reaction constants for the aldehydes studied. together with the bibliographic values obtained for the parameter σ. Figure S1. Influence of [pHBZH] (left) and [HOONO] (right) upon the initial rate at pH = 2. I = 0.1 M and T = 25 °C. Figure S2. Influence of [pMeBZH] (left) and [HOONO] (right) upon the initial rate at pH = 2. I = 0.1 M and T = 25 °C. Figure S3. Influence of [pMoxBZH] (left) and [HOONO] (right) upon initial rate at pH = 2. I = 0.1 M and T = 25 °C. Figure S4. Influence of [oHBZH] (left) and [HOONO] (right) upon the initial rate at pH2. I = 0.1 M and T = 25 °C. Figure S5. Influence of [pTFBZH] (left) and [HOONO] (right) upon the initial rate at pH2. I = 0.1 M and T = 25 °C. Figure S6. Influence of pH upon initial reaction rate [RBZH] = 4.00 × 10^−5^ M; [ONOO−] = 8.00 × 10^−5^ M; I = 0.1 M; Tª = 25 °C. Figure S7: Comparison of the 1H-NMR spectrum obtained from the liquid fraction vs. the 1H-NMR spectrum of benzyl alcohol. 1H-NMR spectrum of benzyl alcohol. Figure S8: Comparison of ^1^H-NMR spectrum obtained from the liquid fraction vs. ^1^H-NMR spectrum of benzyl alcohol. ^1^H-NMR spectrum of benzoic acid. Reaction mechanism in acid and basic media. Figure S9: Graphical representation of the Hammett equation for k_4_ nucleophilic attack. Also, the theoretical rate equation deduction according to has been included [37,38].
