# Supplementary material for: Oxidation of Aldehydes Used as Food Additives by Peroxynitrite
Source: Antioxidants (Basel). 2023 Mar 17;12(3):743. doi: 10.3390/antiox12030743 (PMC10241184; doi:10.3390/antiox12030743)
Supplement: Supplementary file 1 [file antioxidants-12-00743-s001.zip › antioxidants-2218395-supplementary.pdf]

Table S1: Initial rates of all aldehydes in both media; pH=2; pH=11.2 I=0.1M; T=25 ºC.

Benzaldehyde

$v_{0_{BZH}}$ ×10^6 (M/s) pH=2		[Benzaldehyde] ×10^5 (M)				
		1	2	4	6	8
[HOONO] ×10^5 (M)	4	1.64±0.03	1.80±0.04	1.91±0.04	2.02±0.04	2.16±0.04
	6	2.46±0.04	2.70±0.03	2.86±0.02	3.04±0.03	3.24±0.04
	8	3.29±0.03	3.60±0.03	3.82±0.03	4.05±0.02	4.32±0.03
	10	4.11±0.04	4.50±0.03	4.77±0.03	5.06±0.02	5.40±0.03
	20	8.22±0.04	8.99±0.03	9.54±0.04	10.12±0.05	10.81±0.04

Benzaldehyde

$v_{0_{BZH}}$ ×10^9 (M/s) pH=11.2		[Benzaldehyde] ×10^5 (M)				
		1	2	4	6	8
[$ONOO^-$] ×10^5 (M)	4	6.07±0.03	1.19±0.01	1.63±0.02	1.90±0.02	2.08±0.01
	6	9.90±0.02	1.38±0.01	2.18±0.03	2.58±0.01	2.83±0.01
	8	1.05±0.01	1.72±0.01	2.47±0.02	3.92±0.03	5.81±0.02
	10	1.45±0.02	2.21±0.01	4.45±0.03	5.68±0.02	6.47±0.01
	20	2.72±0.01	4.93±0.01	7.28±0.01	9.23±0.01	1.30±0.02

p-hydroxybenzaldehyde

$v_{0_{p-HBZH}}$ ×10^6 (M/s) pH=2		[p-Hydroxybenzaldehyde] ×10^5 (M)				
		1	2	4	6	8
[HOONO] ×10^5 (M)	4	2.38±0.03	3.32±0.03	4.58±0.03	4.85±0.03	4.66±0.03
	6	3.07±0.03	4.41±0.03	6.08±0.04	6.94±0.04	6.93±0.04
	8	3.50±0.03	5.26±0.03	7.54±0.04	8.86±0.05	9.02±0.04
	10	3.99±0.03	5.75±0.03	8.11±0.03	9.82±0.05	10.32±0.04
	20	8.20±0.03	11.09±0.04	13.91±0.04	14.99±0.04	15.52±0.04

p-methylbenzaldehyde

$v_{0_{p-MeBZH}}$ ×10^6 (M/s) pH=2		[p-Methylbenzaldehyde] ×10^5 (M)				
		1	2	4	6	8
[HOONO] ×10^5 (M)	4	2.35±0.02	4.18±0.01	5.75±0.01	7.55±0.02	8.35±0.02
	6	4.20±0.01	6.10±0.01	7.70±0.01	9.90±0.01	10.05±0.01
	8	5.50±0.01	7.20±0.01	10.45±0.02	11.65±0.01	12.45±0.01
	10	7.85±0.01	9.50±0.01	13.31±0.02	14.45±0.02	14.90±0.01
	20	10.80±0.02	14.30±0.01	18.90±0.02	22.00±0.02	24.40±0.01

p-methoxybenzaldehyde

$v_{0_{p-MeOBZH}}$ ×10^6 (M/s) pH=2		[p-Methoxybenzaldehyde] ×10^5 (M)				
		1	2	4	6	8
[HOONO] ×10^5 (M)	4	1.91±0.02	2.87±0.02	4.10±0.03	4.41±0.02	4.48±0.03
	6	2.42±0.02	3.44±0.02	5.09±0.02	5.79±0.03	5.83±0.03
	8	2.80±0.02	3.83±0.02	6.09±0.03	6.99±0.02	7.54±0.03
	10	3.25±0.02	4.90±0.03	6.61±0.03	8.13±0.03	8.53±0.03
	20	4.47±0.03	6.53±0.03	9.46±0.02	10.90±0.03	12.57±0.03

Table S1: (continuation)

p-nitrobenzaldehyde

$v_{0_{p-NBZH}}$ ×10^6 (M/s) pH=2		[p-Nitrobenzaldehyde] ×10^5 (M)				
		1	2	4	6	8
[HOONO] ×10^5 (M)	4	1.24±0.04	1.60±0.02	1.66±0.02	1.74±0.03	1.78±0.04
	6	1.86±0.01	2.39±0.01	2.49±0.02	2.63±0.02	2.68±0.04
	8	2.47±0.01	3.19±0.01	3.30±0.02	3.53±0.02	3.55±0.03
	10	3.09±0.01	4.00±0.02	4.15±0.02	4.11±0.02	4.48±0.04
	20	6.18±0.02	7.98±0.02	8.31±0.03	8.78±0.02	8.97±0.06

p-trifluoromethylbenzaldehyde

$v_{0_{p-TFBZH}}$ ×10^6 (M/s) pH=2		[p-Trifluoromethylbenzaldehyde] ×10^5 (M)				
		1	2	4	6	8
[HOONO] ×10^5 (M)	4	1.01±0.04	1.18±0.04	1.19±0.04	1.21±0.04	1.26±0.04
	6	1.49±0.04	1.83±0.04	1.77±0.04	1.81±0.04	1.89±0.04
	8	1.95±0.04	2.38±0.04	2.37±0.04	2.40±0.04	2.52±0.04
	10	2.47±0.04	2.87±0.04	3.05±0.04	3.12±0.04	3.15±0.04
	20	4.95±0.04	5.60±0.04	6.01±0.04	6.12±0.04	6.31±0.04

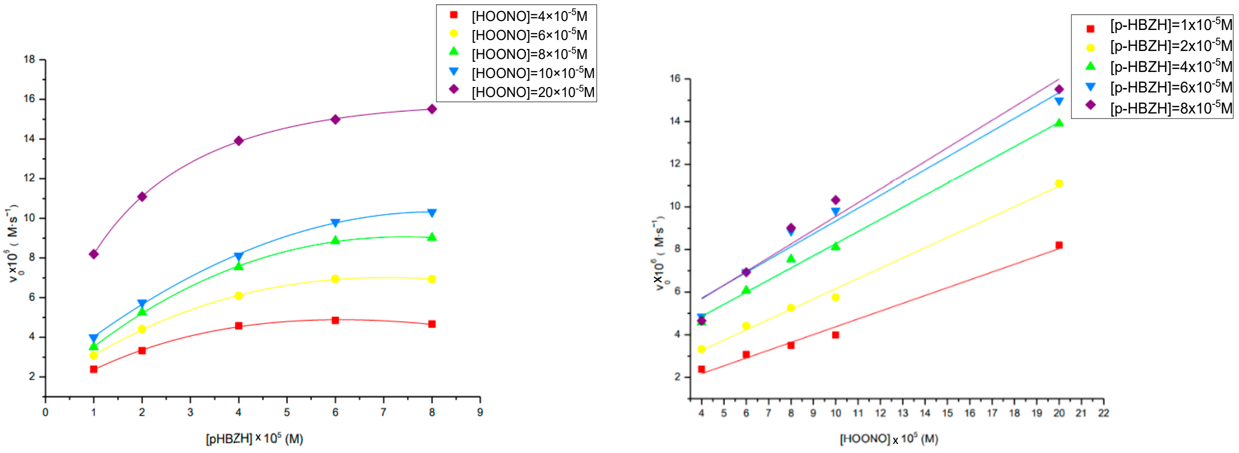

Figure S1. Influence of [pHBZH] (left) and [HOONO] (right) upon the initial rate at pH2. I=0.1M and T=25ºC

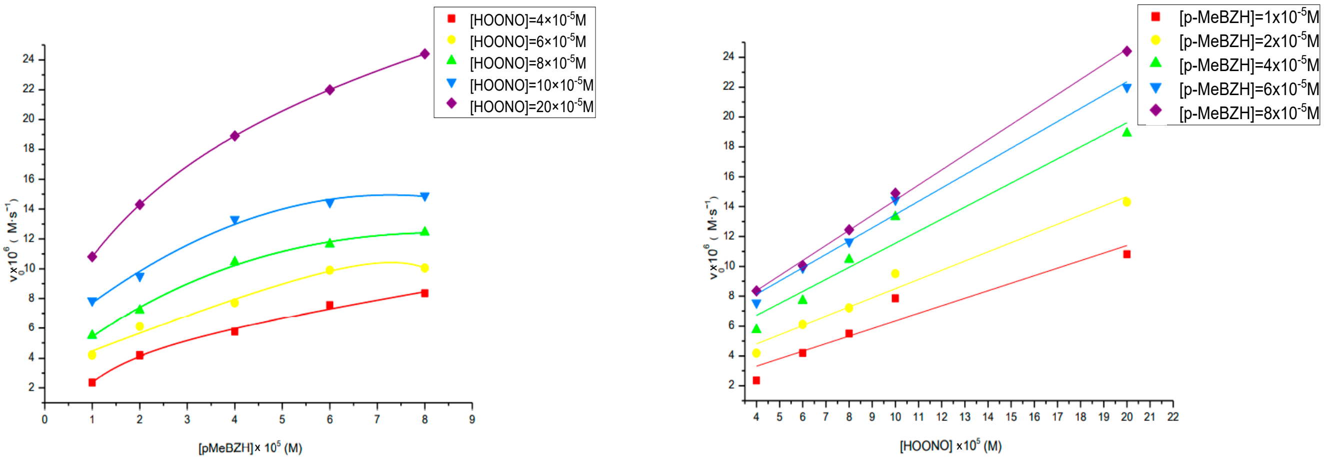

Figure S2. Influence of [pMeBZH] (left) and [HOONO] (right) upon the initial rate at pH2. I=0.1M and T=25ºC

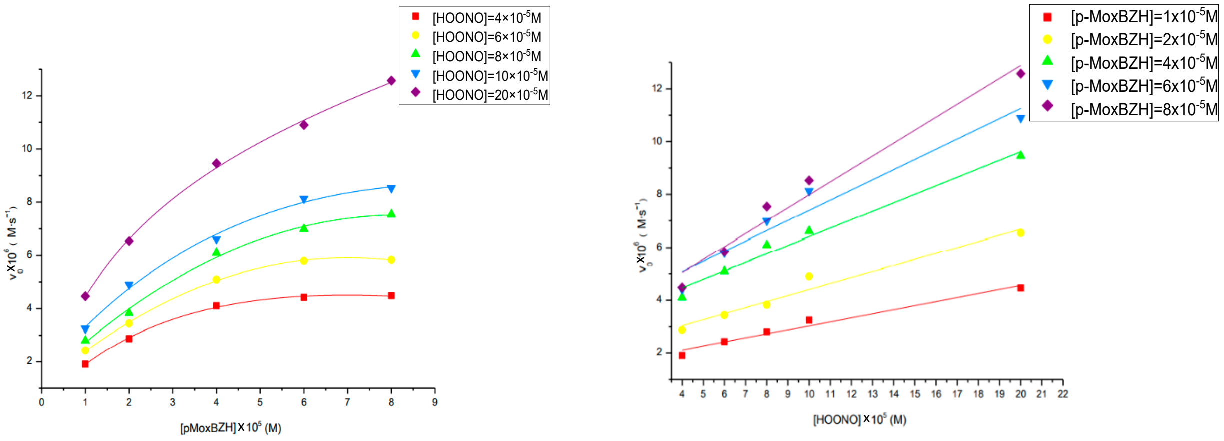

Figure S3. Influence of [pMoxBZH] (left) and [HOONO] (right) upon the initial rate at pH2. I=0.1M and T=25ºC.

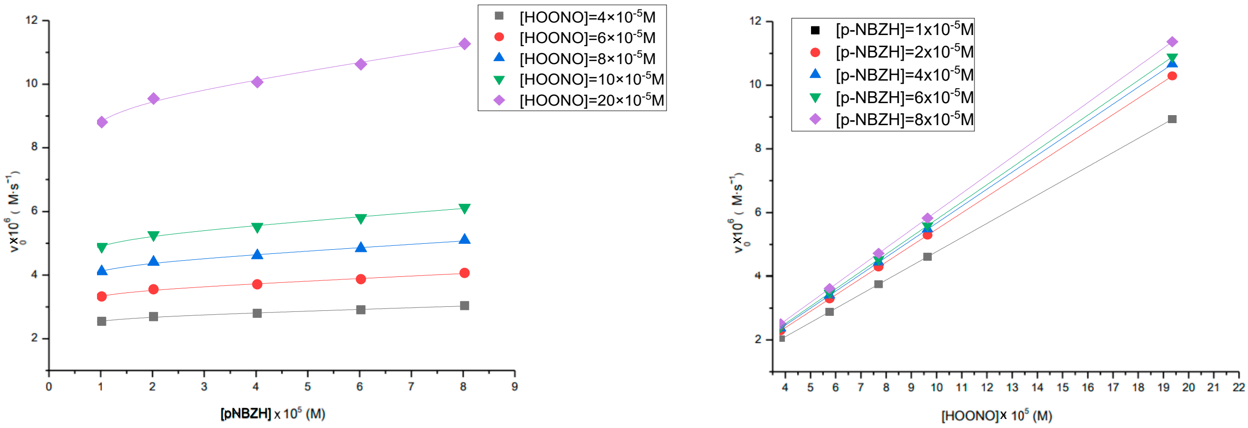

Figure S4. Influence of [pNBZH] (left) and [HOONO] (right) upon the initial rate at pH2. I=0.1M and T=25ºC

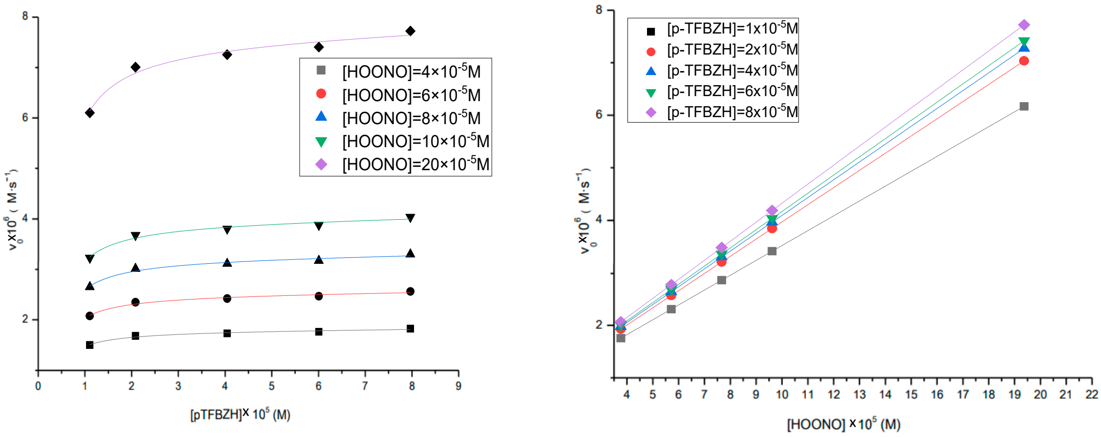

Figure S5. Influence of [pTFBZH] (left) and [HOONO] (right) upon the initial rate at pH2. I=0.1M and T=25ºC

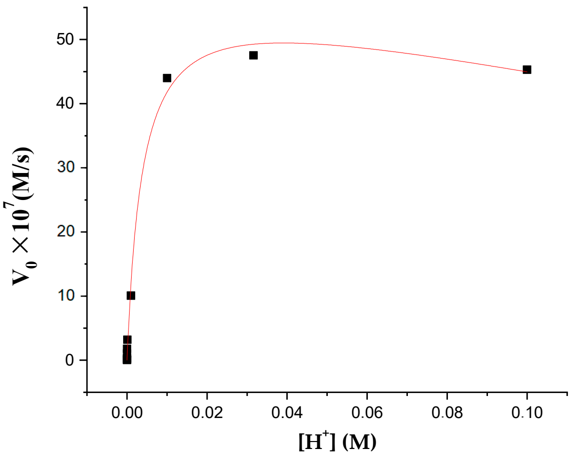

Figure S6. Influence of pH upon the initial reation rate [RBZH]=4.00×10^{-5} M; [ONOO^{-}]=8.00×10^{-5}M; I=0.1M; Tª=25ºC

Table S2: Influence of pH on the initial reaction rate. [RBZH]=4.00×10^{-5} M; [ONOO^{-}]=8.00×10^{-5}M; I=0.1M; Tª=25ºC

**pH**	**v_o×10^7 (M/s)**	**pH**	**v_o×10^7 (M/s)**
12	0.02 ± 0.01	5	1.77 ± 0.01
11	0.02 ± 0.01	4	3.19 ± 0.03
10	0.03 ± 0.01	3	10.07 ± 0.02
9	0.04 ± 0.02	2	38.24 ± 0.01
8	0.15 ± 0.01	1.5	38.45 ± 0.02
7	0.28 ± 0.03	1	38.31 ± 0.01
6	1.03 ± 0.01		

Table S3: Initial velocity values obtained at different ionic strength values at pH=11.20 and pH=2. [BZH]=4.00×10^{-5} M; [ONOO^{-}]=8.00×10^{-5}M.

**pH=11.20**				
**I (M)**	0.5	0.3	0.1	0.05
**v_o×10^9 (M/s)**	2.89±0.03	2.32±0.01	2.46±0.02	2.40±0.02
**pH=2**				
**I (M)**	0.3	0.2	0.1	0.05
**v_o×10^6 (M/s)**	3.08±0.01	3.20±0.03	3.82±0.02	3.18±0.03

Table S4: Influence of temperature on the initial rate *[HOONO] = 8.00×10^{-5} M. [BZH] = 4.00×10^{-5} M. I= 0.1M. pH = 2.*

**Temperature (K)**	**283**	**288**	**293**	**298**	**303**
**v_0×10^6 (M·s^{-1})**	0.82±0.02	1.52±0.02	2.41±0.02	3.82±0.02	5.27±0.02

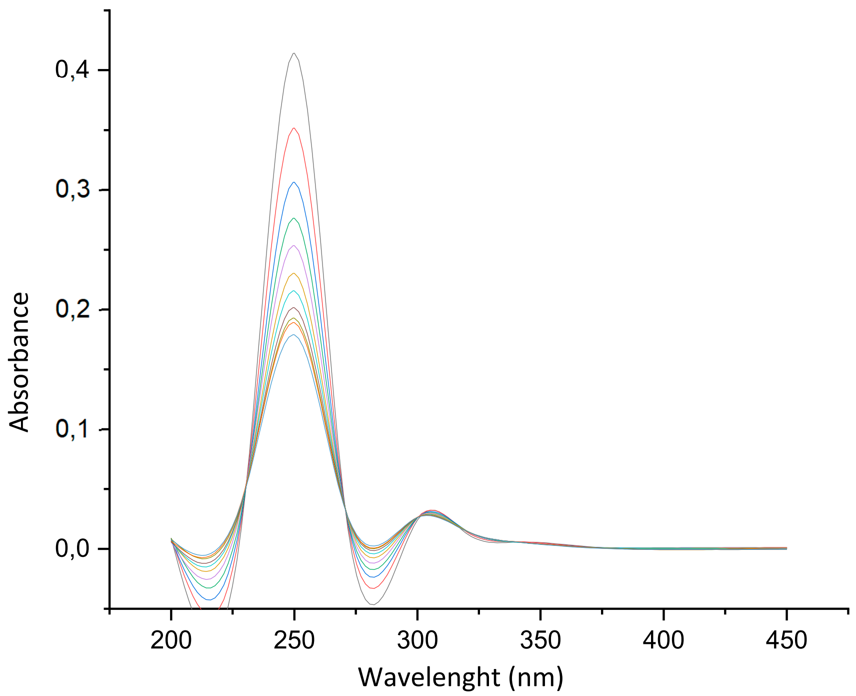

Figure S6: BZH and HOONO reaction spectrum. [BZH]=4.00×10^{-5} M. [HOONO]=8.00×10^{-5} M. pH=2. I=0.1M. T=25 ºC. Total time reaction t=2 s, Δt=0.2 s.

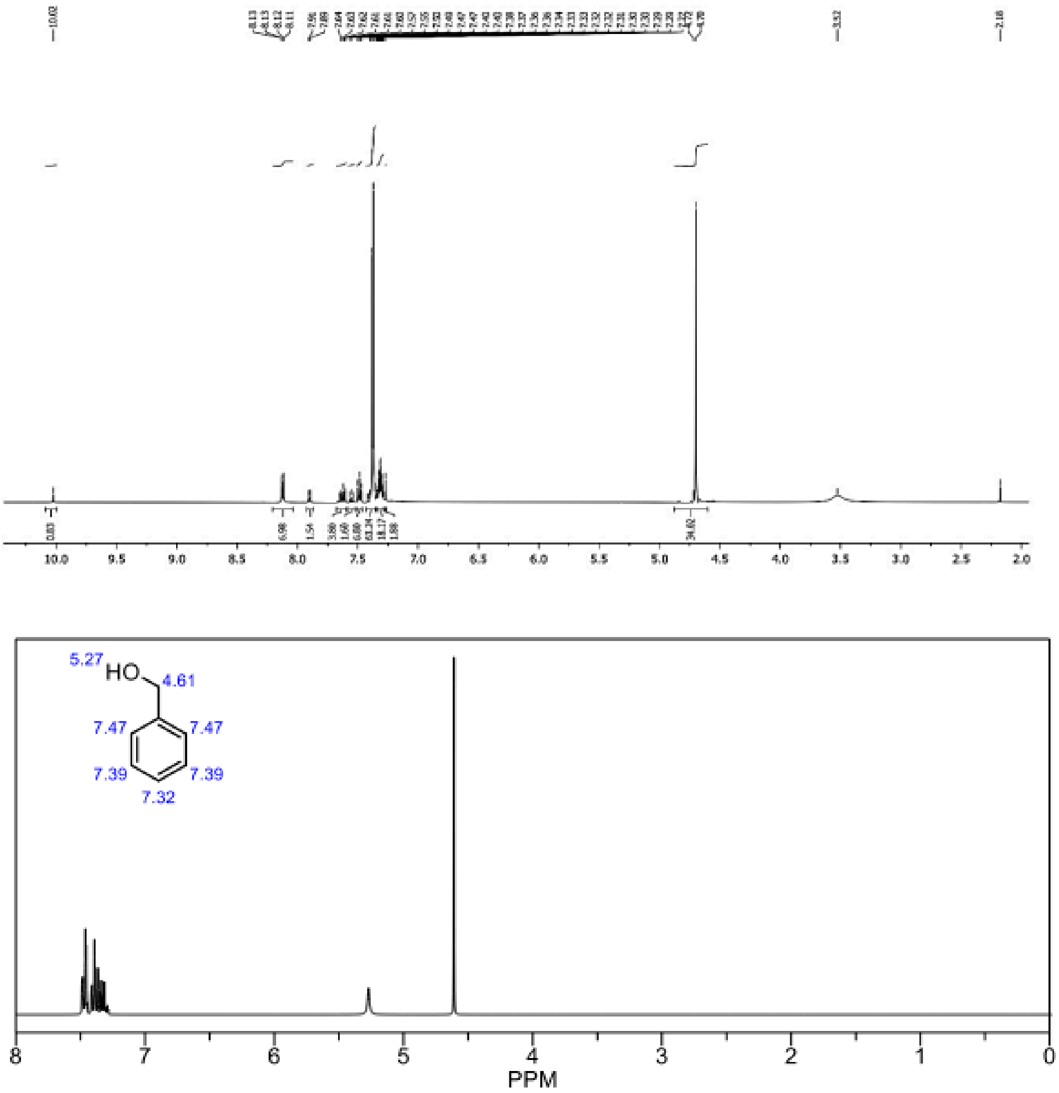

Figure S7: Comparison of the ^{1}H-NMR spectrum obtained from the liquid fraction vs. the ^{1}H-NMR spectrum of benzyl alcohol. ^{1}H-NMR spectrum of benzyl alcohol.

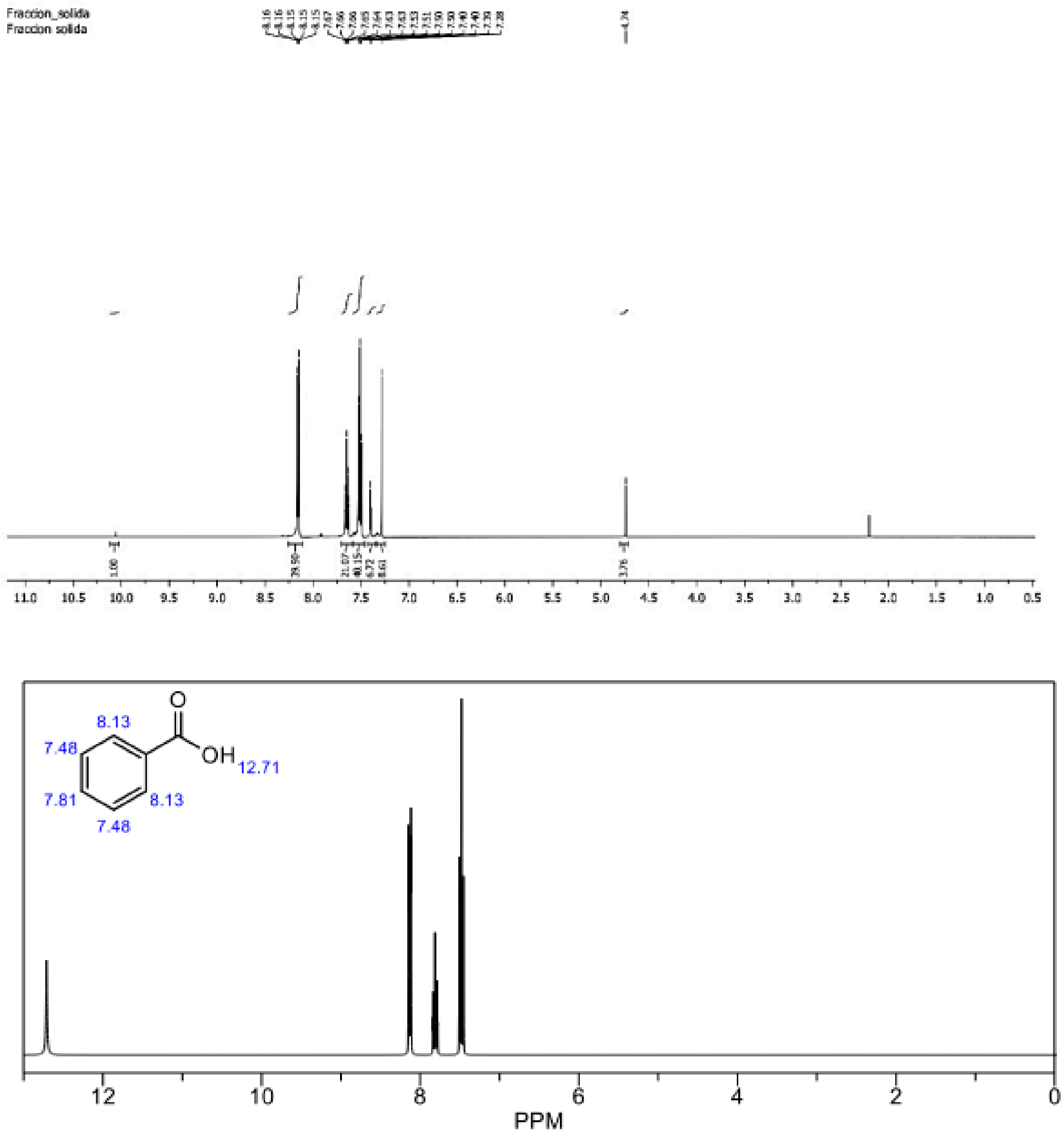

Figure S8: Comparison of the ^{1}H-NMR spectrum obtained from the liquid fraction vs. the ^{1}H-NMR spectrum of benzyl alcohol. ^{1}H-NMR spectrum of benzoic acid.

Deduction of theoretical rate equation according with Squeme 1 on the article

$$v_0 = -\frac{d[RBZH]}{dt} = \{k_2[RBZH^+][\cdot OH] + k_2[RBZH^+][\cdot NO_2]\} + k_3[RBZH][ONOO^-] + \{k_4[RBZH^+][HOONO] + +k_5[RBZH^+][X]\}$$

If the equilibrium 1 and 2 are considered it can write:

$$[ONOO^-] = \frac{K_a[HOONO]}{[H^+]}$$

$$[RBZH^+] = \frac{[RBZH][H^+]}{K_{a'}}$$

Substitute [BZH^+] and [$ONOO^-$] and considered that [$\cdot$OH]=[$\cdot NO_2$]=1/2[Rad] on the theoretical rate equation:

$$v_0 = \frac{d[RBZH]}{dt} = 2k_2[RBZH^+][Rad] + k_3[RBZH]\left(\frac{K_a[HOONO]}{[H^+]}\right) + \frac{k_4[RBZH][H^+]}{K_{a'}}[HOONO] + \frac{k_5[RBZH][H^+]}{K_{a'}}[X]$$

Applying the steady-state approximation to [X]

$$\frac{d[x]}{dt} = 0 = \frac{k_4[RBZH][H^+]}{K_{a'}}[HOONO] - \frac{k_5[X][RBZH][H^+]}{K_{a'}} - k_6[X]$$

$$[X] = \frac{k_4[RBZH][H^+][HOONO]}{k_5[RBZH][H^+] + k_6K_{a'}}$$

Take in account a mass balance equation for the peroxynitrite concentration.

$$[HOONO]_0 = [HOONO] + [ONOO^-]$$

$$[HOONO] = [HOONO]_0\left(\frac{[H^+]}{[H^+] + K_a}\right)$$

$$[HOONO] = [HOONO]_0\ A$$

So [X] is:

$$[X] = \frac{k_4[RBZH][H^+][HOONO]_0\,A}{k_5[RBZH][H^+] + k_6K_{a\prime}}$$

Replacing [HOONO] and [X] on the theoretical rate equation:

$$v_0 = \frac{d[RBZH]}{dt} = 2k_2[RBZH^+][Rad] + k_3[RBZH]\left(\frac{K_a[HOONO]_0A}{[H^+]}\right) + \frac{k_4[RBZH][H^+]}{K_{a\prime}}[HOONO]_0A + \frac{k_5[RBZH][H^+]}{K_{a\prime}}\frac{k_4[RBZH][H^+][HOONO]_0\,A}{k_5[RBZH][H^+] + k_5K_{a\prime}}$$

The final theoretical rate equation is:

$$v_0 = (2k_2[RBZH^+][Rad]) + \left(\frac{k_3[RBZH]K_a[HOONO]_0A}{[H^+]}\right) + \left(\frac{k_4[RBZH][H^+][HOONO]_0A}{K_{a\prime}}\right) + \left(\frac{k_5k_4[RBZH]^2[H^+]^2[HOONO]_0A}{k_5[RBZH][H^+] + k_6K_{a\prime}^2}\right)$$

The only constants we can calculate are $k_{3.}$ $k_{4.}$ k_5 $_{\text{and}}$ k_6

At pH 11.2 the initial reaction rate was modified by a factor 0.98 (98%) in order to obtain k_3. direct oxidation by peroxynitrite anion.

Knowing: $[H^+]=10^{-11}$. $K_a=10^{-6.8}$ [37]

$$v_{oxb} = \frac{k_3[RBZH]K_a[HOONO]_0\left(\frac{[H^+]}{[H^+] + K_a}\right)}{[H^+]}$$

Linear adjustment:

$$v_0 = k_3[RBZH]$$

$$k_3 = \frac{\text{slope}[H^+]}{K_a(\frac{[H^+]}{[H^+] + K_a})}$$

$$k_3 = 7.17 \times 10^{-1}M^{-1}s^{-1}$$

The experimental rate equations is:

$$v_0 = E[RBZH] + F$$

Where:

$$E = \frac{k_3[HOONO]_0 K_a A}{[H^+]}$$

In order to obtain k_4 and k_5/k_6 the initial rate is connected by a factor of 0.64 (64%) at pH 2. Knowing: $[H^+]=10^{-2}$. $K_a=10^{-6.8}$

Table S5: pK'a values for each of the studied benzaldehydes.

Aldehyde	$-pK_{a'}$ [38]
BZH	7.10
pHBZH	4.35
pMeBZH	6.32
pMeOBZH	5.54
pNBZH	8.45
pTFBZH	7.97

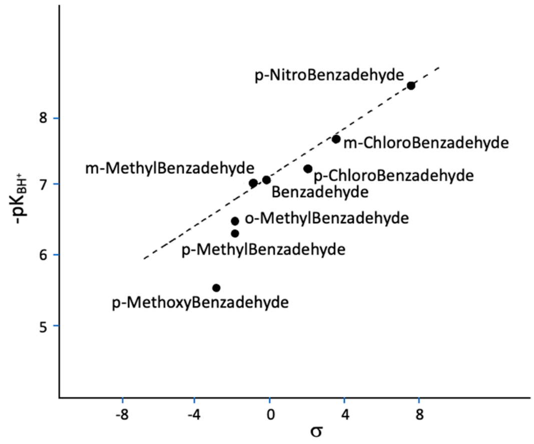

$$v_{0\,Cn} = \frac{k_4[RBZH][H^+]A}{k'_a} + \frac{k_5 k_4 [RBZH]^2[H^+]^2 A}{k'_a(k_5[RBZH][H^+] + k_6 k'_a)}[HOONO]_0$$

$$v_{0\,Cn} = \frac{k_5 k_4[RBZH]^2[H^+]^2A + k_4 k_6 k'_a[RBZH][H^+]A + k_4 k_5[RBZH]^2[H^+]^2A}{k'_a(k_5[RBZH][H^+] + k_6 k'_a)}[HOONO]_0$$

$$v_{0\,Cn} = \frac{2k_5 k_4[RBZH]^2[H^+]^2A + k_4 k_6 k'_a[RBZH][H^+]A}{k'_a k_5[RBZH][H^+] + k_6 {k'_a}^2}[HOONO]_0$$

$$\frac{v_0}{[HOONO]_0} = \frac{A[RBZH]^2 + B[RBZH]}{C[RBZH] + D}$$

$$A = 2k_5[H^+]k_4A$$
$$B = k_6k'_ak_4[H^+]A$$
$$C = k_5k'_a[H^+]$$
$$D = k_6{k'_a}^2$$

Rational adjustment:

$$v_0 = \frac{A[RBZH]^2 + B[RBZH]}{C[RBZH] + D}$$

Table S6: Parameters of the rational fit and reaction constants obtained for each aldehyde.

**Aldehydes**	**A×10^{-8}**	**B×10^{-4}**	**C×10^{-6}**	**D**	**r**
BZH	1.53±0.13	5.18±0.27	1.8±0.04	1.69±0.02	0.99398
pHBZH	51.5±0.32	4.80±0.12	1.41±0.09	11.53±0.08	0.99816
pMeBZH	4.86±0.25	7.07±0.09	1.57±0.73	7.55±0.08	0.99435
pMeOBZH	2.91±0.04	1.58±0.19	1.18±0.03	6.56±0.02	0.99462
pNBZH	36.0±0.02	5.13±0.09	1.57±0.28	9.10±0.06	0.96615
pTFBZH	10.8±0.14	3.23±0.02	1.58±0.12	4.22±0.03	0.96951

So replacing A.B.C.D in:

$$A = 2k_5[H^+]k_4A$$
$$B = k_6k'_ak_4[H^+]A$$
$$C = k_5k'_a[H^+]$$
$$D = k_6{k'_a}^2$$

Table S7: Reaction constants for the aldehydes studied. together with the bibliographic values obtained for the parameter σ

**Aldehyde**	**k_4×10^{-3} ($M^{-1}s^{-1}$)**	**k_5 ($M^{-1}s^{-1}$)**	**k_6×10^{+14}(s^{-1})**	**σ**
BZH	24.37±1.21	14.29±0.71	1.06±0.05	0.00
pHBZH	1861.42±93.07	6298.23±314.91	2300000±115000	-0.92
pMeBZH	44.86±2.24	75.14±3.75	172±8.6	-0.17
pMoxBZH	69.53±3.47	340.31±17.01	5450±272.5	-0.27
pNBZH	0.20±0.01	0.56±0.02	0.01±0.0005	0.78
pTFBZH	0.82±0.04	1.69±0.08	0.05±0.002	0.54

The others experimental rate equations are:

$$v_0 = D[HOONO]_0$$
$$v_0 = G[ONOO^-]_0$$

$$v_0 = \frac{J[H^+] + K[H^+]^2}{1 + L[H^+]}$$

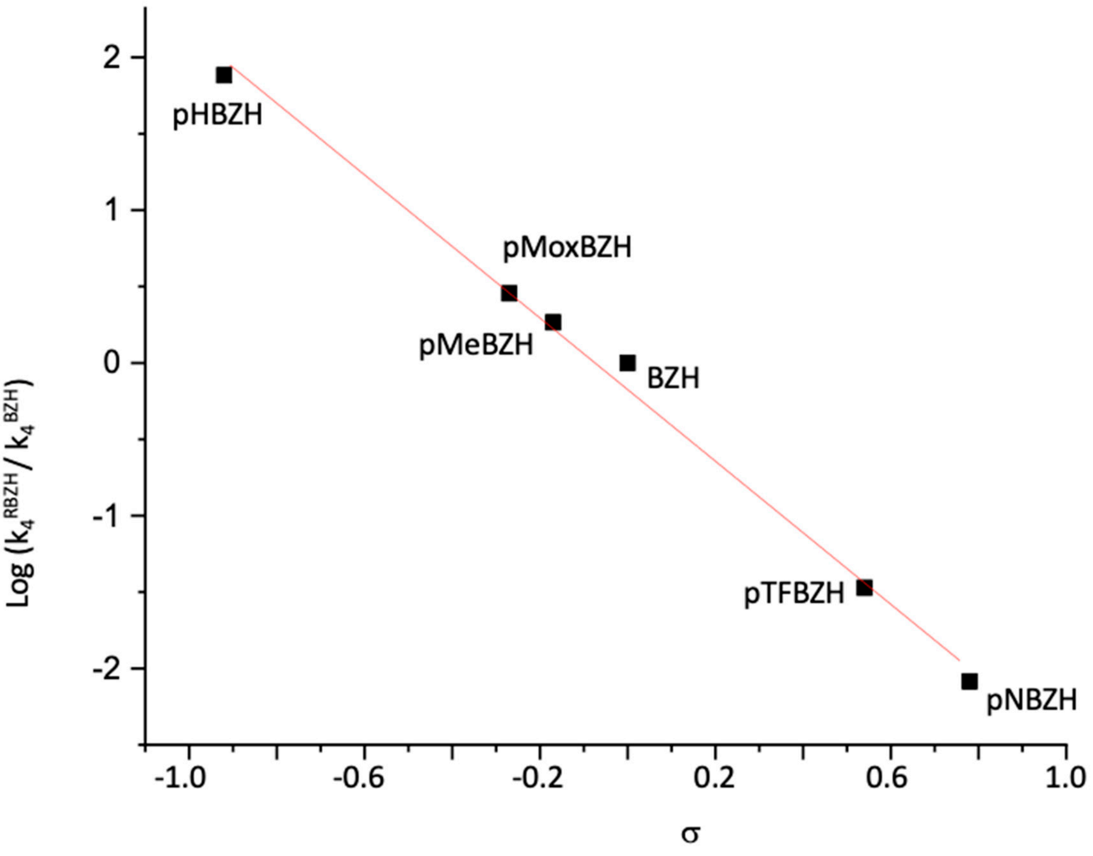

Figure S9: *Graphical representation of the Hammett equation for k_4* nucleophilic attack.
